# Fluvastatin Interferes with Hepatitis C Virus Replication via Microtubule Bundling and a Doublecortin-like Kinase-Mediated Mechanism

**DOI:** 10.1371/journal.pone.0080304

**Published:** 2013-11-19

**Authors:** Naushad Ali, Heba Allam, Ted Bader, Randal May, Kanthesh M. Basalingappa, William L. Berry, Parthasarathy Chandrakesan, Dongfeng Qu, Nathaniel Weygant, Michael S. Bronze, Shahid Umar, Ralf Janknecht, Sripathi M. Sureban, Mark Huycke, Courtney W. Houchen

**Affiliations:** 1 Department of Medicine, Section of Digestive Diseases and Nutrition, University of Oklahoma, Oklahoma City, Oklahoma, United States of America; 2 Department of Cell Biology, University of Oklahoma, Oklahoma City, Oklahoma, United States of America; 3 Peggy and Charles Stephenson Cancer Center, University of Oklahoma Health Sciences Center, University of Oklahoma, Oklahoma City, Oklahoma, United States of America; 4 Department of Veterans Affairs Medical Center, University of Oklahoma, Oklahoma City, Oklahoma, United States of America; 5 Department of Molecular and Integrative Physiology, and Medicine, University of Kansas Medical Center, Kansas City, Kansas, United States of America; 6 Department of Microbiology and Immunology, National Liver Institute, Menoufiya University, Menoufiya, Egypt; Rosalind Franklin University of Medicine and Science, United States of America

## Abstract

Hepatitis C virus (HCV)-induced alterations in lipid metabolism and cellular protein expression contribute to viral pathogenesis. The mechanism of pleiotropic actions of cholesterol-lowering drugs, statins, against HCV and multiple cancers are not well understood. We investigated effects of fluvastatin (FLV) on microtubule-associated and cancer stem cell marker (CSC), doublecortin-like kinase 1 (DCLK1) during HCV-induced hepatocarcinogenesis. HCV replication models, cancer cell lines and normal human hepatocytes were used to investigate the antiviral and antitumor effects of statins. FLV treatment resulted in induction of microtubule bundling, cell-cycle arrest and alterations in cellular DCLK1 distribution in HCV-expressing hepatoma cells. These events adversely affected the survival of liver-derived tumor cells without affecting normal human hepatocytes. FLV downregulated HCV replication in cell culture where the ATP pool and cell viability were not compromised. Pravastatin did not exhibit these effects on HCV replication, microtubules and cancer cells. The levels of miR-122 that regulates liver homeostasis and provides HCV genomic stability remained at steady state whereas DCLK1 mRNA levels were considerably reduced during FLV treatment. We further demonstrated that HCV replication was increased with DCLK1 overexpression. In conclusion, unique effects of FLV on microtubules and their binding partner DCLK1 are likely to contribute to its anti-HCV and antitumor activities in addition to its known inhibitory effects on 3-hydroxy-3-methylglutary-CoA reductase (HMGCR).

## Introduction

HCV is a positive strand RNA virus classified as a hepacivirus of the family Flaviviridae. The viral infection leads to chronic hepatitis in the majority of patients (>80%) and often progresses to cirrhosis or hepatocellular carcinoma [1], [2]. HCV genomic RNA encodes a single polyprotein that is processed co-translationally into three structural (C-E1-E2) and seven nonstructural (p7-NS2-NS3-NS4A-NS4B-NS5A-NS5B) polypeptides. HCV induces web-like membranous structures and uses lipid-rafts and microtubule filaments (MTFs) for its replication via negative strand synthesis [3], [4].

Current triple drug therapy for HCV infection consists of a NS3 protease inhibitor (telaprevir or boceprevir), pegylated interferon-α and ribavirin (Peg-IFN/RBV). Although, rapid viral response (RVR) and sustained viral response (SVR) are improved with this regimen, cure in a large group of patients remains an unmet medical need. It has been suggested that host genetic factors such as IFNL3/4 alleles, [5] socio-economic and pre-existing health conditions, adverse effects of the drugs, and emergence of viral genetic variants are associated with resistance to current HCV treatment [6]. Inhibitors of NS5A and NS5B proteins are in various stages of clinical development [7], [8].

Dose studies by Bader et al. [9] and worldwide randomized controlled trials [10]–[14] show that fluvastatin significantly improves HCV treatment outcome and that the FDA-approved dosages of fluvastatin are well tolerated by patients with chronic HCV infection. However, beneficial effects of FLV in certain patients are contested by other studies [15] and warrants further investigations on the mechanism of FLV-mediated inhibition of HCV replication. Statins are inhibitors of HMGCR, which catalyzes a rate-limiting reaction in cholesterol biosynthesis and converts HMG-CoA to mevalonic acid. These drugs are used for the treatment of hypercholesterolemia and have been reported to exhibit activities against viruses and cancer cells [16]. The precise mechanism of the anti-HCV activities of statins is undefined. Recent studies suggest that statins inhibit geranylgeranylation of FBL2, which is critical for the interaction of FBL2 with NS5A and HCV replication [17], [18]. It has not been demonstrated that the degree of inhibition of FBL2 differs between the available statins. Statins vary in the degree of anti-HCV activity, with pravastatin having no activity at all [19]. From prospective randomized controlled trials with fluvastatin, it is clear that changes in serum lipids do not correlate with anti-HCV activity. Inclusion of fluvastatin improves SVR during PEG-IFN/ribavirin therapy for patients with high viral loads [11].

We previously documented that liver-derived hepatoma cells express high levels of tumor/cancer stem cell (CSC) markers such as Myc, CD133, α-fetoprotein and doublecortin-like kinase (DCLK1, also called DCAMKL-1) in response to HCV replication [20]. DCLK1 has also been recognized as a CSC marker in intestine, colon and pancreas [21]–[23]. In addition, we showed overexpression of DCLK1 in the hepatic pre-neoplastic nodules of HCV patients and a positive correlation between DCLK1 expression and HCV replication [20]. The protein has been shown to associate and catalyze polymerization of microtubules [24], which are required for the movement of HCV replication complexes and the viral replication [25], [26]. Thus, lipid metabolism [27], [28] and stem cell-related proteins contribute to HCV pathogenicity [20]. The susceptibility of hepatic progenitor cells for HCV infection and persistent viral replication in these cells [29] also support this concept. Newer HCV inhibitors that simultaneously target the infection as well as HCV-induced pathological changes in liver may improve clinical status, enhance anti-viral efficacies and reduce the risk of drug-resistance. Here, we present evidence of a novel mechanism of fluvastatin action on HCV-expressing cells. Besides inhibition of cholesterol biosynthesis, FLV disrupts DCLK1-microtubule axis, which adversely affects HCV replication and cancer cell survival.

## Materials and Methods

### Cell culture and transfection

The GS5 cells used in these studies are derived from hepatoma Huh7.5 cell line and express HCV-1b subgenomic replicons encoding for NS5A-GFP [NS5A containing green florescence protein (GFP) at C-terminus] [30]. The intensity of GFP approximately correlates with the extent of HCV replication and most GFP in these cells is present as the full-length chimeric NS5A-GFP protein. The FCA4 cells are derived from Huh7 hepatoma cell line and express a similar subgenomic replicon but lack GFP tag with NS5A. The HCV replication in this cell line has been characterized previously [31], [32]. The cells were maintained at 60–80% confluency level during experiments and as described earlier [20]. The plasmids pJFH1 and pJFH1/GND encode wild type and inactive NS5B polymerase in the full-length HCV-2a RNA respectively [33]. The DNAs were linearized with XbaI and transcribed with T7 RNA polymerase (Promega). The synthesized RNAs were purified, checked for integrity and transfected into Huh7.5 cells. The cryopreserved normal human hepatocytes (NHHs) were purchased from BD Biosciences. For immunofluorescence microscopy, the NHHs were cultured/maintained in Hepato-STIM Hepatocyte Defined Medium (BD Biosciences) supplemented with epidermal growth factor (10 ng/ml) and 2 mM L-glutamine on collagen cover slips. The cells were incubated at 37°C and 5% CO2. Alternatively, NHH were also maintained in Matrigel 6-well plate in the Hepato-STIM media for spheroid assay.

#### Real-time reverse transcription-PCR

The real-time reverse transcription-PCR analyses were carried out using total RNA as described earlier [20]. Total RNAs were isolated from control (untreated), DMSO or FLV treated cells using RNeasy isolation kit (Qiagen). The RNA samples were analyzed for purity/integrity and subjected to reverse transcription with Superscript II and random hexanucleotide primers (Invitrogen). In the subsequent step, the cDNAs were used as templates to perform real-time PCR by SYBR chemistry method (SYBR® Green I; Molecular Probes). The target (HCV, DCLK1, miR-122) and control (actin) RNAs were amplified using Jumpstart Taq DNA polymerase (Sigma) and the following primers:

*Actin*: 5′GGTGATCCACATCTGCTGGAA-3′(forward)

5′ATCATTGCTCCTCCTCAGGG3′(reverse);

*DCLK1*: 5′AGTCTTCCGATTCCGAGTTGAG3′(forward);

5′CAGCAACCAGGAATGTATTGGA3′(reverse);

*HCV*: 5′CGGGAGAGCCATAGTGG3′(forward)

5′AGTACCACAAGGCCTTTCG3′(reverse).

*miR-122:* 5′CCTTAGCAGAGCTGTGGAGTG3′(forward)

5′GCCTAGCAGTAGCTATTTAGTG3′(reverse)

The crossing threshold values assessed by the real-time PCR were evaluated for the transcripts. Actin mRNA level in each sample served as an internal control. The mRNA levels were expressed as fold change relative to control with ± SEM value. The HCV RNA level in untreated cells was considered as one (or 100% HCV RNA level). The levels of HCV RNAs in DMSO or FLV-treated cells were compared with the untreated sample in real-time PCR.

### Treatment of cells with statins and cell proliferation assay

The cells were treated with varying amounts (0.5 µM through 10 µM) of fluvastatin (FLV, Reference Standard, USP Rockville, MD) dissolved in dimethyl sulfoxide (DMSO) for 48 and 72 h. For each FLV treatment, corresponding amounts (μl) of DMSO were added in similar cell culture as a control. Various concentrations of mevalonic acid (Sigma, Mev) were added as antagonist of FLV in similar cultures. Pravastatin was dissolved in water and its corresponding treatment control was supplemented with same amount of water. Cell viability and ATP assays were carried out using commercial kits (Promega).

### DCLK1 overexpression

The red fluorescence protein (RFP) coding sequences were cloned in-frame at the 5′ end of DCLK1 (NM_004734) ORF to generate RFP-DCLK1 cassette in pENTR-DsRedEx2 vector (Addgene). Using clonase, the cassette was transferred to pLenti-CMV-PURO-Dest plasmid (gift from Dr. Eric Campeau) to generate pLenti-RFP-DCLK1 plasmid vector. For untagged DCLK1 expression, the ORF was cloned into pLenti-CMV-Puro-Dest plasmid. The expression plasmid was packaged into lentivirus by transient plasmid transfection of 293T cells together with the three helper plasmids (pMD2.G, pMDL/RRE g/p, and pRSV-REV). The viral particles in the supernatants were concentrated. Following infection, the cells were selected for 7–10 days in the presence of puromycin (10 µg/ml). The resistant cells were further grown under normal culture conditions without puromycin. A control expression vector (pLenti-RFP) expresses only RFP and was packaged into viruses as described above. Total lysates of cultured cells were prepared using M-PER lysis buffer (Pierce) and Western blots were carried out by chemiluminescence method (GE Healthcare). The primary antibodies against HCV NS5B, DCLK1, actin and RFP (all purchased from Abcam) were used for the detection of respective protein bands. To evaluate target protein to actin ratio, the band intensities were calculated using Gelquant software.

#### Immunofluorescence and confocal microscopy

Cells grown on glass cover-slips (VWR) were rinsed briefly in phosphate-buffered saline (PBS), fixed in 4% paraformaldehyde in PBS pH 7.4 for 20 min at room temperature, washed twice with ice cold PBS and permeabilized in ice-cold acetone. Cells were incubated with blocking buffer (10% serum, 0.01% Triton X-100, in PBS, pH 7.4) for 1 hour, washed with PBS and treated with anti-DCLK1 (Abcam) and/or anti-α-tubulin (Santa Cruz) antibodies in PBS-T containing 1% BSA for 1–2 hr at room temperature or overnight at 4°C. After thorough washing with PBS-T, cover-slips were incubated in appropriate AlexaFluor conjugated secondary antibodies. The nuclei were counterstained with DAPI (0.1–1 µg/ml PBS). The cover-slips were mounted on microscope slides in ProLong Gold antifade reagent (Invitrogen) for detection of Immunofluorescence using Nikon 80i or Leica TCS NT (for confocal microscopy).

### Florescence-activated cell sorting (FACS) and cell cycle analysis

The cultured cells were trypsinized and suspended in phosphate buffer saline (PBS) containing paraformaldehyde (2%) for 1 h at 4°C. The cells were washed twice with ice-cold PBS, resuspended in PBS containing 70% ethanol, and incubated overnight at 4°C. The cells were washed and resuspended in PBS for the treatment with RNase A (200 µg/ml) and propidium iodide (PI, 50 µg/ml) (Sigma) for 30 min at 37°C in the dark. The treated cells were stored at 4°C until FACS analysis. The instrument was calibrated with unstained cells and sorted for PI and GFP intensities.

## Results

### Anti-HCV activities of fluvastatin (FLV) in replicon-expressing GS5 cells

**Figure 1:**
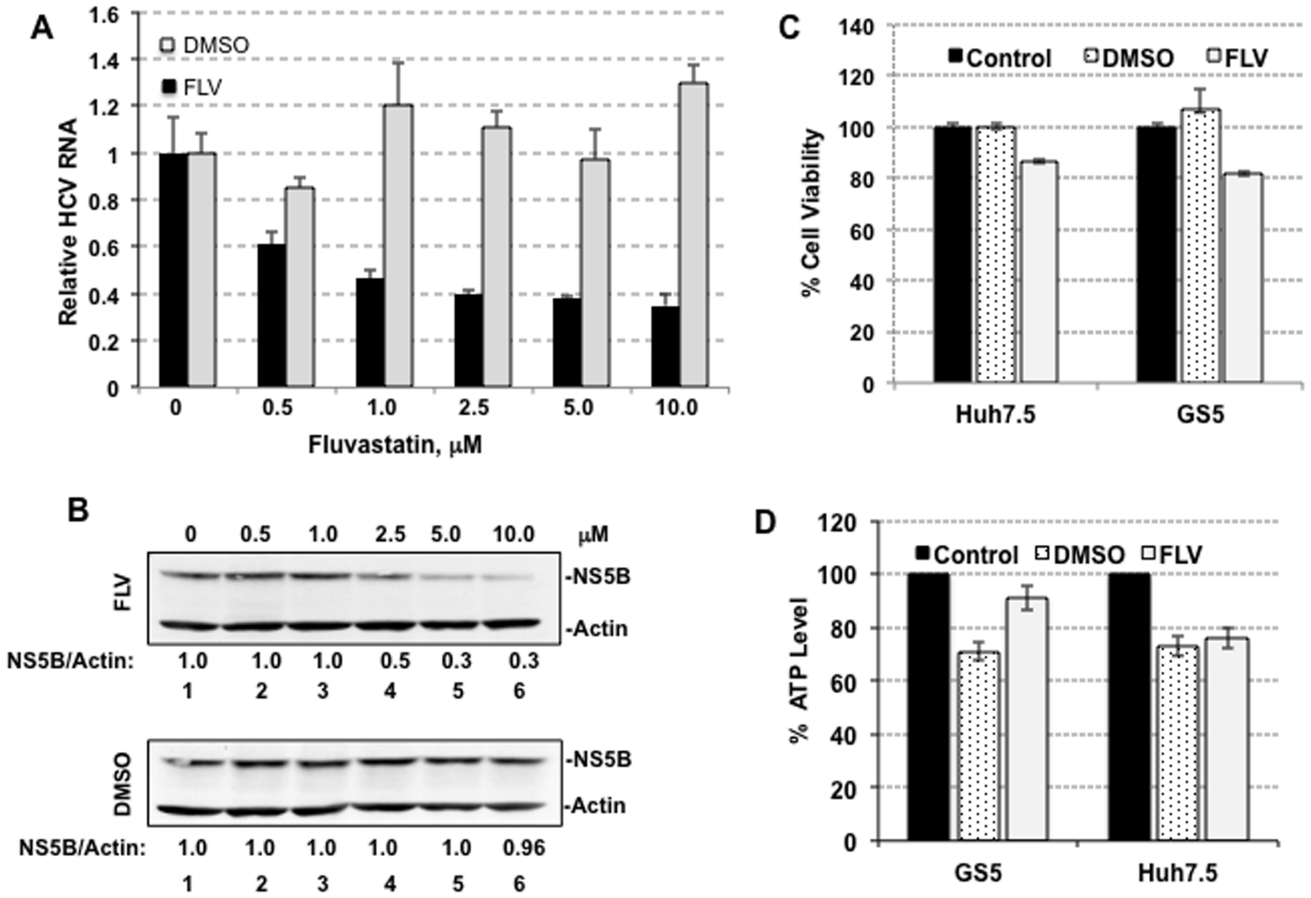
Inhibition of HCV replication by fluvastatin (FLV) in cell culture. (A) Real-time RT-PCR for detection of the HCV RNA in DMSO (control, gray bar) or FLV (black bar) treated GS5 cells. These cells were first isolated by FACS using GFP channel, expanded and treated with varying amounts (0–10 µM) of FLV or similar amounts of DMSO for 48 hr. The relative HCV RNA levels in treated cells were measured in total RNA extracted and compared with untreated cells (zero control). (B) Western blot for detection of NS5B levels in FLV (lane 2 through 6, upper panel) or DMSO (lower panel) treated cells. Lane 1, untreated control. Actin, loading control. Band intensities of NS5B and actin were calculated and NS5A/actin ratio in untreated control was set as one. (C) Determination of cytotoxicity of FLV (5 µM) or DMSO for Huh7.5 and GS5 cells using cell viability assay. Control, untreated sample. (D) ATP assay for detection of metabolic status of GS5 and Huh7.5 cell lines following FLV (5 µM) or DMSO treatment. Five thousand each of the cell lines were plated in a 96-well opaque walled tissue cultures plate and ATP level was measured using luciferase-based assay kit (Promega). Each treatment was carried out in triplicates. ATP levels in the DMSO- or FLV-treated cells were compared with those of untreated (black bar, considered as 100% ATP level) cells.

The HCV subgenomic replicon-expressing GS5 cells were treated with varying concentrations of FLV (0.5 µM through 10 µM) for 48 hr. These cellular concentrations of fluvastatin mimic the known pharmacologic range of fluvastatin when prescribed for humans [9]. The cell cultures were maintained at 60%–80% confluency during treatments with FLV or vehicle DMSO. Total RNAs isolated from the cultured cells were subject to real-time PCR for the detection of positive-strand HCV RNA. Actin mRNA in each case served as an internal control. The HCV RNA levels in DMSO or FLV samples were compared with the untreated level that was set as one (Fig. 1A). Within 48 h, a dose-dependent reduction (maximum 60–70%) in viral RNA was observed in the FLV-treated cells, but not in the DMSO treated cells. The maximum inhibition was achieved at 5–10 µM of FLV. The reduction in HCV RNA in the FLV-treated cells correlated with the decrease in the NS5B polymerase (Fig. 1B, upper panel, lanes 3–6) whereas the polymerase levels were unaffected in the DMSO-treated samples (lower panel). Cell viability assay suggests that most GS5 or Huh7.5 (control cells that lack replicon) cells were alive when treated with 5 µM of FLV (Fig. 1C). Only a minor decrease in cell viability was observed in these samples as compared to DMSO treated or untreated controls. Similarly, ATP assay suggests that both GS5 and Huh7.5 cells were metabolically active in the presence of 5 µM FLV during treatment (Fig. 1D). We used these treatment conditions for subsequent investigations.

### Specificity of Fluvastatin-mediated downregulation of HCV replication

**Figure 2:**
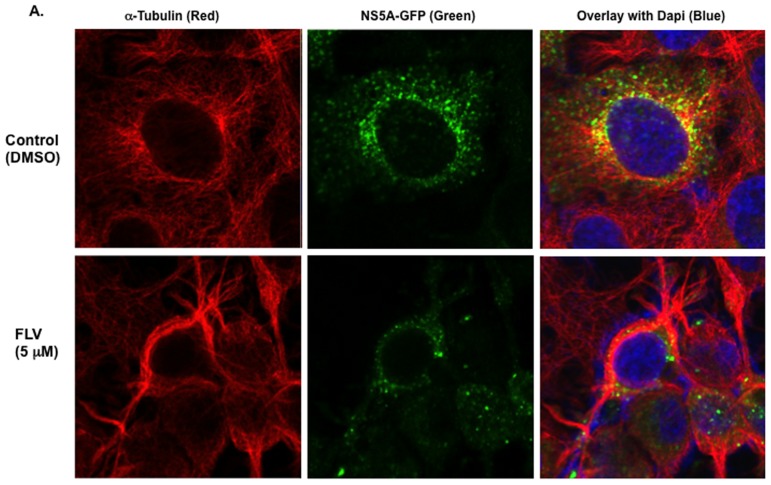
Specificity of anti-HCV effects of fluvastatin. (A) Real-time RT-PCR for detection of HCV RNA in DMSO (control, gray bar) or FLV (black bar) or pravastatin (Prav, hatched bar) treated FCA4 cells. The cells were treated with varying amounts (0, 2.5 or 5.0 µM) of the drugs. Similar amounts of DMSO were used in the corresponding controls. The relative HCV RNA levels were measured in total RNA extracted from the treated cells and compared to the untreated samples (set at one for RNA level). Actin mRNA for each sample is used as internal control. Each sample was run in triplicate for PCR and experiments were repeated three times. (B) Western blot for detection of NS5B protein levels (upper panel) in DMSO (lanes 2, 3), FLV (lanes 4, 5) or Prav (lanes 6, 7) treated FCA4 cells. Lane 1, untreated control. Actin, loading control (lower panel). (C) Determination of FCA4 cell viability using MTT assay after treatment with 5 µM FLV or DMSO for 48 hr. Huh7 (replicon negative hepatoma cells and ASPC1, an unrelated pancreatic cell lines were used as additional controls. (D) Normal human hepatocytes cultured on collagen I-coated plate were treated with varying amounts (0 through 10 µM) of FLV or Prav. DMSO was used as control for each FLV-treatment. The cells were subjected to viability assay. The statins-treated cells were compared with the untreated cells (considered as 100% viable). (E) In vitro transcribed infectious JFH1 RNA was transfected into Huh7.5 cells. Following 6 hr of transfection, the cells were treated with DMSO (lane 2) or 5 µM FLV (lane 3) for 48 hr and the lystaes were subjected to Western blot for detection of NS5B. Lane 1, untreated JFH1-expressing cells (control).

To address whether observed FLV effects on HCV is not restricted to GS5 cells (derived from a single Huh7.5 clone), we used FCA4 cell line that is derived from original Huh7 hepatoma cells (heterogeneous population) and expresses a subgenomic replicon (HCV-1b) but without GFP tag [31], [32]. The HCV RNA was significantly reduced at both FLV concentrations (2.5 and 5.0 µM) to varying degrees when compared to the untreated samples (Fig. 2A). In contrast, HCV RNA levels in DMSO and pravastatin (Prav)-treated samples remained similar to the untreated controls. The specific inhibitory effect was further reflected in Western blot analyses that showed considerable reduction in the NS5B level only in the FLV-treated cells (Fig. 2B, lanes 4, 5) but not in the control (lanes 2, 3) or Prav-treated cells (lanes 6, 7). The FCA4 cell viability remained unaffected after treatment with 5 µM FLV (Fig. 2C) during this period. Two other cell-types, AsPC1 (pancreatic cancer cell line) and Huh7 showed minor sensitivity to the drug during similar experiments. The monolayer culture of normal human hepatocytes on collagen 1-coated dish did not exhibit cytotoxicity to FLV or Pra up to 5 µM (Fig. 2D).

Because the subgenomic replicons lack structural proteins, we transfected Huh7.5 cells with *in vitro* synthesized full length JFH1 RNA that is known to produce HCV infectious particles [33]. Six hours following transfection, cells were treated with DMSO or FLV for 48 h and NS5B levels were measured by Western blot. As shown in Fig. 2E, FLV treatment (lane 3) resulted in a considerable decrease in the NS5B polymerase as compared to the controls (lanes 1, 2). Thus, the inhibitory effect of FLV on the HCV expression is not limited to the GS5 cells but rather is similarly observed in different cells of hepatic origin and with the full length infectious HCV RNA.

### Fluvastatin induces microtubule bundling in hepatoma cells but not in normal human hepatocytes

**Figure 3:**
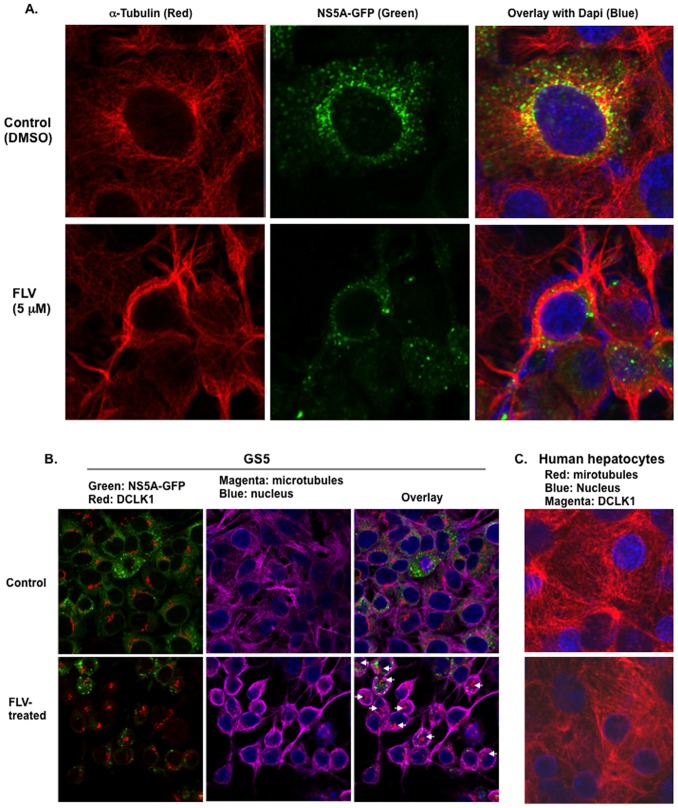
Immunoflorescence and Confocal microscopy revealed FLV-induced microtubule bundling in hepatoma cells but not in normal human hepatocytes. (A) Fluvastatin-induced microtubule bundling affects the HCV NS5A levels and its cytoplasmic distribution. GS5 cells were stained for α-tubulin (red) and nucleus (blue) after treatment with DMSO (upper panel, control) or 5 µM FLV (lower panel) for 48 hr. HCV NS5A-GFP (green) and colocalization of NS5A-GFP with microtubules (yellow, overlay images) are shown. (B) Relative localization of DCLK1, NS5A and microtubules. GS5 cells treated with DMSO- (control, upper panel) or 5 µM FLV (lower panel) prior to immunoflorescence staining. Green, NS5A-GFP; red,DCLK1; magenta, α-tubulin. White arrows indicate poor attachment of DCLK1 to microtubules. (C) Normal human hepatocytes (NHH) stained negative for DCLK1 (magenta-negative) and lacked FLV-induced microtubule (red) bundling (lower panel). Microtubules (red) are distributed throughout the cell body in both DMSO- and FLV- treated NHHs.

The morphological changes observed in GS5 or FCA4 (not shown) cells following treatment with FLV and the known association of DCLK1 protein with microtubules [24] prompted us to investigate the impact of FLV treatment on the cellular cytoskeleton using confocal microscopy. When GS5 cells were treated with DSMO alone, NS5A-GFP (green) and/or its complexes indicated as punctate green dots [26] showed association with microtubules (red) in the perinuclear regions (Fig. 3A, green and yellow dots in the upper panel on right side). However, FLV-treated GS5 cells exhibited extensive ‘bundling’ of the microtubules (lower panel, red) again mostly perinuclear. This effect was accompanied by significant loss of NS5A-GFP and induction of round-shaped cell morphology (lower panel). Co-staining of similar GS5 cultures (Fig. 3B, upper panel) revealed that cellular DCLK1 (red) is distributed in the area rich in NS5A-GFP (green dots) and microtubules (magenta). However, FLV-induced microtubule bundling results in altered localization of DCLK1 and poor attachment with microtubules (lower panel, indicated with arrows in overlay image), additionally, NS5A-GFP expression is suppressed/reduced following FLV treatment (lower panel, green). Similar FLV effects were also observed on the parent Huh7.5 cells that lack HCV replicon (not shown). In contrast, human hepatocytes derived from normal liver were completely resistant to the FLV-induced microtubule bundling (Fig. 3C, lower panel, microtubules-red and DCLK1-megenta) and these cells lacked detectable DCLK1 staining in both FLV-treated and control cells. Besides GS5 and Huh7.5 hepatoma cells, colon (HCT116) and pancreatic (AsPC1) cancer cell lines also exhibited similar morphological perturbation (not shown). Confocal microscopy of these cells confirmed FLV-induced microtubule bundling and DCLK1 dislocation from the microtubule filaments (data not shown).

**Figure 4:**
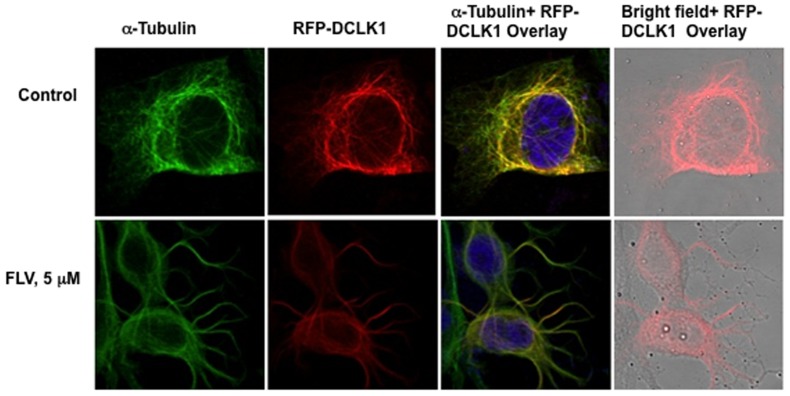
Effects of fluvastatin on DCLK1 overexpressing hepatoma cells. Upper panel; α-tubulin was highlighted with Alexa Flour 647 (far red)-secondary antibody conjugate staining but pseudocolored as green in GS5 cells overexpressing red florescence protein (RFP) tagged-DCLK1 (RFP-DCLK1, red). Yellow, colocalization of RFP-DCLK1 with microtubules. Lower panel; the distribution of microtubule (green) and DCLK1 (red) in 5 µM FLV-treated RFP-DCLK1 cells. *Far right*, bright field image of the cells was overlaid with DCLK1 (red) to demonstrate morphological changes and re-distribution of DCLK1 into the bundled microtubules after FLV treatment. Most DCLK1 overexpressing cells showed similar distribution pattern as shown here.

Because DCLK1 is frequently overexpressed in pre-neoplastic and cancer tissues [20], [22], we investigated FLV effects on the GS5-DCLK1 cells, which overexpress human DCLK1 tagged with red florescence protein (RFP) at the N-terminus. The microtubule distribution is highlighted by staining with α-tubulin in these cells (Fig. 4, green, upper panel). Extensive colocalization (Pearson coefficient ∼ 0.8) of the chimeric DCLK1 (red) was observed with microtubules in the perinuclear regions (yellow color). The FLV treatment resulted in disruption of normal microtubule architecture with unusual protrusion or neurite-like growth (lower panel, Pearson coefficient ∼ 0.4). Unlike GS5 cells, these cells did not exhibit aggregates of DCLK1 but the protein was distributed along with bundled microtubules. We also noticed overall reduction in the DCLK intensities following FLV-treatment. Other cell lines expressing DCLK1 showed similar MTF bundling following FLV treatment (data not shown). Thus, these results clearly demonstrate a positive correlation between MTF-associated DCLK1 and HCV replication and sensitivity of DCLK-overexpressing cells to FLV treatment.

### DCLK mRNA but not miR-122 is downregulated by fluvastatin

**Figure 5:**
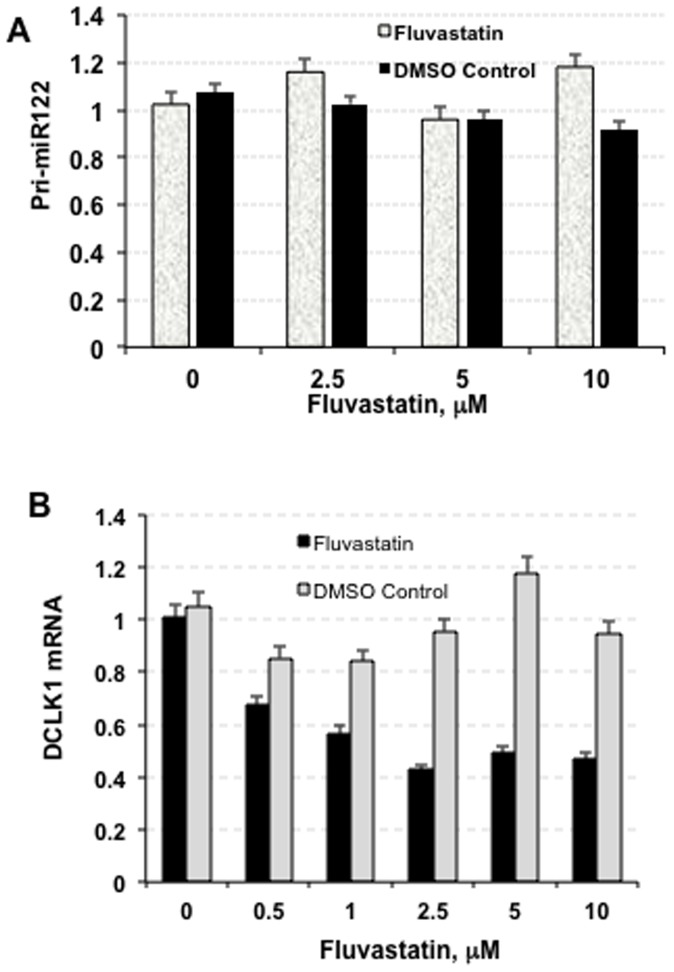
Fluvastatin downregulates DCLK1 mRNA level (B) but not the abundance of miR-122 (A). The GS5 cells were treated with varying amounts of FLV (as indicated) or similar amount of the solvent (DMSO). Total RNAs isolated from these cells were subjected to real-time RT-PCR. The RNA level in the cells without FLV or DMSO was considered as one and relative RNAs were calculated in treated samples as described in the legend to Fig. 1.

The binding of a liver-enriched miR-122 at two adjacent sites within the 5′ proximal region of the HCV 5′UTR (5′ untranslated region) results in the recruitment of an Ago2-containing RISC-like complex [34]. These interactions physically stabilize the viral RNA genome and promote HCV replication [35]. In addition, *miR-122* regulates homeostasis and fatty acid metabolism in liver [36]–[38]. For these reasons, we investigated if a change in *miR-122* status contributes to the anti-HCV activities of FLV. As shown in Fig. 5A, the *pri-miR-122* levels were similar in FLV-treated (2.5, 5, 10 µM) or DMSO or untreated GS5 cells. However, DCLK1 mRNA levels were considerably decreased (50–55%) due to FLV treatment of these cells (Fig. 5B). These results suggest that FLV modestly reduces DCLK1 mRNA levels, but does not affect *pri-miR-122* level.

### DCLK1 positively regulates HCV replication

**Figure 6:**
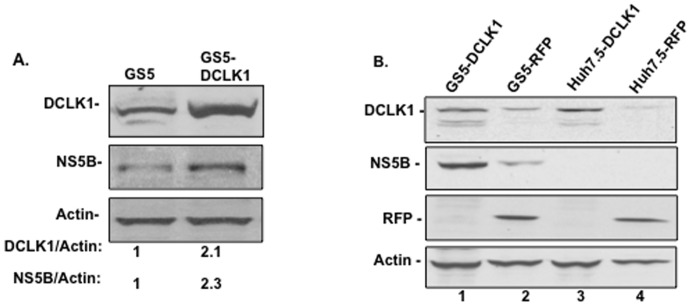
Overexpression of DCLK1 in GS5 cells stimulates HCV expression. (A) Comparison of DCLK1 and NS5B levels in GS5 with that of GS5-DCLK1 (GS5 cells overexpressing untagged DCLK1). The ratio of DCLK1 or NS5B to actin is given below the respective lanes. (B) Levels of DCLK1 and NS5B in GS5 cell lines expressing a nonspecific red florescence protein (GS5-RFP, lane 2) instead of exogenous DCLK1 (lane 1). The Huh7.5-derived cells (control) expressing exogenous DCLK1 (lane 3) or RFP (lane 4) were used as controls. Thirty microgram of each lysate was used for Western blot analysis.

The level of DCLK1 protein and the numbers of DCLK1-positive cells are markedly increased in chronic HCV-infected patients with evidence of cirrhosis and hepatic nodules [20]. We investigated FLV effects on HCV replication in DCLK1 overexpressing cells (GS5-DCLK1). Hepatoma cells (GS5 and Huh7.5) were infected with lentiviruses expressing untagged human DCLK1. Quantitative analysis of protein bands in Western blot assays (DCLK1 or NS5B to actin ratio) suggests that nearly 2-fold increased DCLK1 expression in GS5-DCLK1 cells (Fig. 6A) caused concomitant increase in the NS5B level as compared to the GS5 counterpart. The positive impact of exogenous DCLK1 on HCV was specific because red fluorescence protein (RFP, a nonspecific protein expression control) overexpressing GS5 cells (GS5-RFP) did not exhibit comparable NS5B level (Fig. 6B, compare lane 2 with 1). Huh7.5-DCLK1 (Fig. 6B, lane 3) expressed DCLK1 more than RFP-expressing cells (Huh7.5-RFP, Fig. 6B, lane 4). These observations suggest that the overexpressed DCLK1 exerted its biological effect on HCV and merely a foreign gene expression (e.g. RFP) did not activate endogenous DCLK1 expression.

### NS5A-negative cell population is increased following fluvastatin treatment

**Figure 7:**
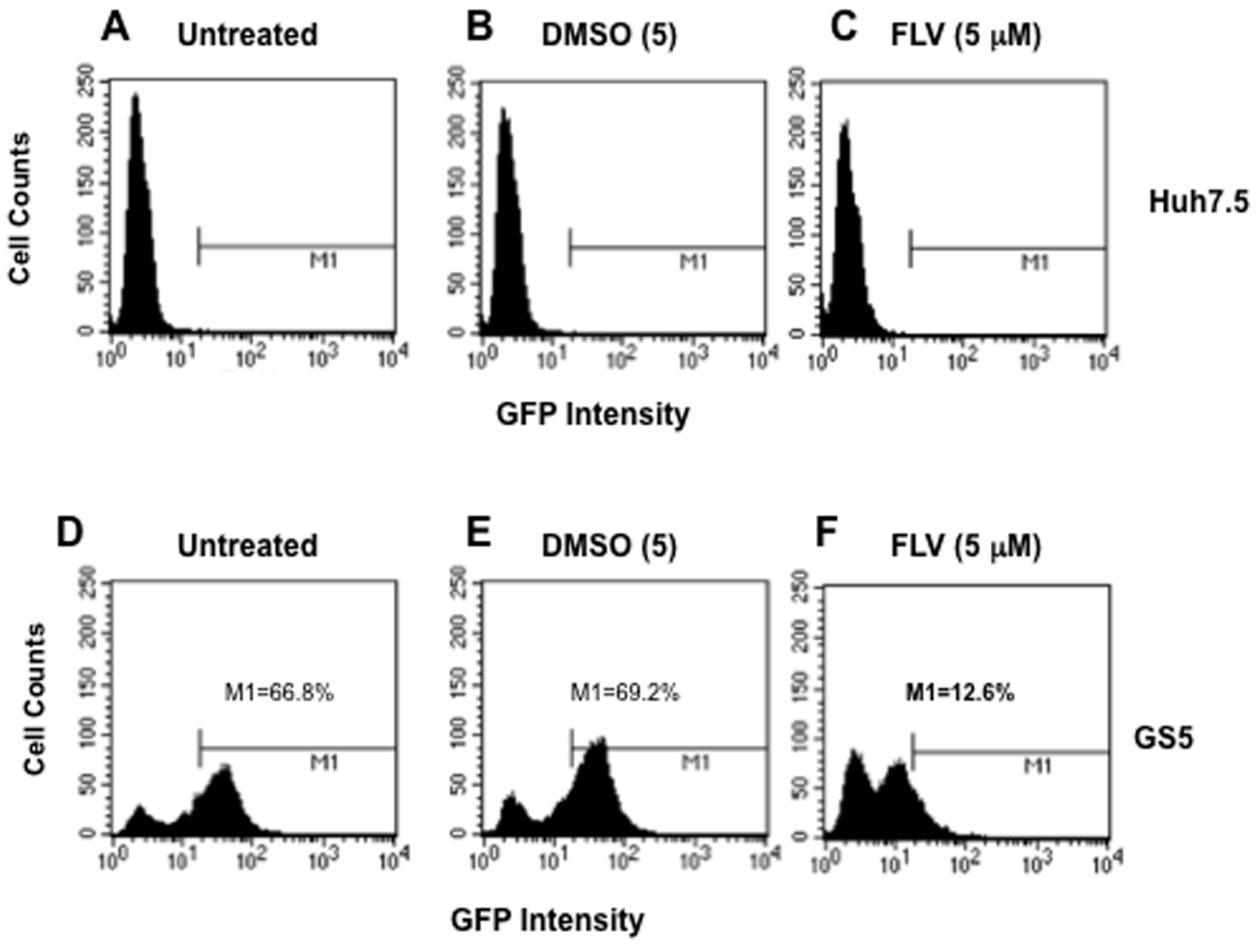
The FLV treatment significantly reduces NS5A-GFP level and increases HCV-negative cell population. The untreated and FLV-treated GS5 cells were subjected to FACS analysis using FITC channel. Approximately 66.8% of total cell populations expressing high levels of NS5A-GFP chimera were gated (M1) in untreated (D), treated with DMSO (E) or FLV(5 µM) (F). The percentage of cells in the gated region is shown for each sample. (A–C), Huh7.5 cells treated as described for GS5 and used for defining HCV-negative cell populations.

In GS5 cells, GFP is expressed as a HCV NS5A hybrid rather than as free protein, and the intensity of GFP in these cells correlates with levels of NS5A and HCV replication [30]. FLV-treated GS5 or Huh7.5 cells were fixed and stained with propidium iodide (PI) to determine NS5A-GFP expression and cell cycle status using FACS method. The instrument was calibrated for GFP-negative cells using Huh7.5 cells prepared under similar FLV-treated and control conditions (Fig. 7A-C). Approximately 80% of the total GS5 cell population was positive for NS5A-GFP (Fig. 7D). The high NS5A-GFP expressing cells (average 67–70%) were gated in the untreated samples (Fig. 7D, indicated as M1) and this region was compared with the histograms of DMSO- and FLV-treated GS5 cells. A significant decrease (∼5–6 fold) in GFP intensity and concomitant increase in the number of GFP-negative cells were observed in the FLV-treated samples (Fig. 7F) as compared to the DMSO control (Fig. 7E).

**Figure 8:**
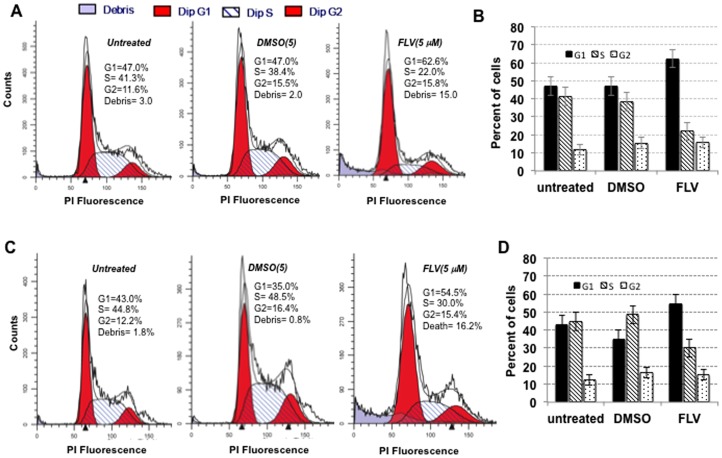
FLV blocks growth of hepatoma cells and causes cell cycle arrest at G1/S phase. The propidium iodide (PI) stained untreated-, DMSO- or FLV-treated GS5 (A, B) or Huh7.5 (C, D) cells were subjected to FACS analysis. The results of three independent experiments showing various stages of cell status in the control and FLV-treated samples for GS5 (B) and Huh7.5 (D) cells are as indicated.

The normal cell cycle progression for GS5 and Huh7.5 cells is shown in Fig. 8A and 8C (untreated panels) respectively. Both cell types showed similar cell cycle patterns after DMSO-treatment. Interestingly, majority of the FLV-treated GS5 (Fig. 8B) and Huh7.5 (Fig. 8D) cells showed cell-cycle status similar to these controls except a modest decrease in S-phase that was accompanied by similar increase in G1 phase. In addition, an average of 15–16% of cells fell under debris category, which reiterated the findings for MTT and ATP assay (Fig. 1C). These observations are also consistent with FLV's known antitumor activities [39] because Huh7 or GS5 cells are tumor-derived and their divisions are impaired by FLV. Thus, majority of cells were alive during the FLV (5 µM)-treatment even though a significant population in the cultures were arrested at G1/S phase.

## Discussion

The inhibitory effect of fluvastatin (FLV) on the cholesterol/mevalonic pathway is very well known and forms the basis of treating hypercholesterolemia with this drug [40]. In addition, lipid-rafts and geranylgeranylation of F-box/leucine-rich repeat protein 2 (FBL-2), that is dependent on the mevalonate pathway, is required for HCV replication [18]. Thus, a strong relationship between HCV replication and lipid metabolism exists and statins are expected to disrupt this association. In spite of lower binding affinity for HMGCR than other statins [41], FLV exhibits anti-HCV activity in patients [9] as well as in the HCV expression models [19], [42]. Ikeda et al. [19] demonstrated that high Mev to FLV ratio (10 mM Mev to 5 µM FLV) could overcome FLV-led inhibition of the HCV replication. We also found a similar rescue effect by Mev in addition to recovery of the cells from morphological alteration (not shown). Based on our analyses and those by others [17], it is highly possible that FLV exerts its anti-HCV influence by disrupting lipid metabolism as well as DCLK1-microtubule axis. FLV treatment of HCV-expressing cells resulted in reduced expression of key viral components (HCV RNA, NS5A and NA5B), disruption of microtubule dynamics, reduction of DCLK mRNA and cell cycle perturbation. In contrast, pravastatin that a potent inhibitor of HMGCR, neither showed anti-HCV activity nor it induced microtubule bundling under similar assay conditions. Although normal human hepatocytes were unaffected by FLV as expected from the clinical use, its deleterious impact on hepatoma and several other cancer cell lines was highly pronounced. The damaging effects of FLV on these cells appear to be attributed to expression and localization of the DCLK1 protein. Interestingly, the levels of miR-122 that regulates cholesterol biosynthesis in liver, stabilizes the viral genome and positively regulates HCV translation/replication, [35] was well maintained during FLV treatment. Thus, the data presented here may explain why statins have markedly different anti-HCV and anti-tumor effects.

Two groups of antitumor drugs that interfere with microtubule dynamics have been described [43], [44]. Microtubule disruptive drugs (vinblastine, nocodazole) suppress microtubule polymerization and induce fragmentation by stimulating microtubule minus-end detachment from their organizing centers [43]. These microtubule-destabilizing drugs affect interaction of NS3 and NS5A with the microtubule and hamper movement of the replication complexes [25], [26]. The second group, the taxanes (paclitaxel, docetaxel), stabilize microtubule polymers and prevent microtubule disassembly. These drugs induce microtubule bundling and create defects in cell division [45]. Fluvastatin appears to induce bundling in cancer cells but not in normal hepatocytes as expected. This difference may be partly attributed to high expression of DCLK1 in cancer cells compared to human normal liver tissues [20] or cultured hepatocytes (Fig. 3). It is possible that HCV replication was impaired due to compromised dynamic instability of MTFs in FLV-treated cells. In this regard, the antiviral and antitumor (because of cell cycle arrest and cell death by >5–10 µM FLV after 48 hr of treatment) activities of FLV appear to be analogous to that of the taxanes.

The siRNA-mediated knockdown of DCLK1 results in inhibition of HCV replication and downregulation of an array of factors that favors tumorigenesis and metastasis [20], [22], [23]. Thus, the microtubule polymerizing activities and MTF association of DCLK1 appears to play certain role in HCV-induced pathogenesis.[20]. Even though fully differentiated NHH lack detectable or high level DCLK1 expression yet HCV is known to replicate in fully differentiated hepatocytes. In our experience, NHH assume DCLK-positive and albumin-negative (DCLK1^+^Alb^−^) phenotype when cultured as spheroids in Matrigel that contains many growth factors. In addition, we have shown that HCV induces DCLK1 and progenitor/stem cell-related markers.[20] Thus, DCLK1 might regulate and/or activate HCV replication even though the basal replication does not require this protein. Our DCLK1 overexpression data favor this possibility (Fig. 6). Wu et al. [29] also demonstrated that albumin-negative hepatic progenitor cells persistently support HCV replication.

In conclusion, these studies suggest that the antiviral effects of FLV may extend beyond its cholesterol-lowering ability. Thus, FLV may be a useful adjunct in the treatment of chronic HCV infection and HCC. FLV exhibits tumor cell selectivity and inhibits HCV without changing the status of miR122, which is an important regulator of liver physiology and the cholesterol pathway [38]. The worldwide-randomized controlled clinical trials of fluvastatin added to peginterferon/ribavirin have demonstrated a significant improvement in cure for HCV genotype 1 compared to the control group of peginterferon/ribavirin alone [46]. These latter results combined with the data presented here offer a potential novel mechanism of action for the beneficial effects of FLV against HCV and HCV-induced liver diseases via interaction with the DCLK1-microtubule axis. Further studies are needed to determine whether FLV in combination with direct acting anti-HCV drugs will improve treatment outcomes.
