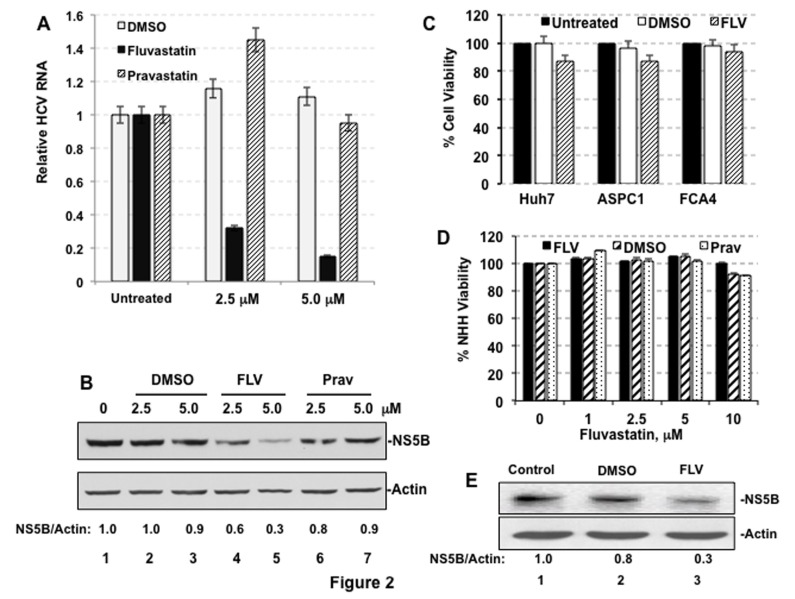# Correction: Fluvastatin Interferes with Hepatitis C Virus Replication via Microtubule Bundling and a Doublecortin-like Kinase-Mediated Mechanism

**DOI:** 10.1371/annotation/fcc840e2-225c-4d71-920d-a1eab7747e19

**Published:** 2013-12-13

**Authors:** Naushad Ali, Heba Allam, Ted Bader, Randal May, Kanthesh M. Basalingappa, William L. Berry, Parthasarathy Chandrakesan, Dongfeng Qu, Nathaniel Weygant, Michael S. Bronze, Shahid Umar, Ralf Janknecht, Sripathi M. Sureban, Mark Huycke, Courtney W. Houchen

Figure 2 is incorrect. The correct version of Figure 2 can be viewed here: 

**Figure:**